# Exploration of Hydroxychloroquine to Improve Perinatal Outcomes in Women with Isolated Non-Specific Auto-Antibody Positivity During Pregnancy

**DOI:** 10.3390/jcm15072758

**Published:** 2026-04-06

**Authors:** Shenglong Ye, Xueqing Zhao, Jinxia Zhao, Yan Wang, Yongqing Wang

**Affiliations:** 1 Department of Obstetrics and Gynecology, Peking University People’s Hospital, Beijing 100044, China; yeshenglong163@163.com (S.Y.); pkuphwangyan@bjmu.edu.cn (Y.W.); 2 Department of Obstetrics and Gynecology, Peking University Third Hospital, Beijing 100191, China; zhaoxueqing1208@163.com; 3 National Clinical Research Center for Obstetrics and Gynecology, Beijing 100191, China; 4 Department of Rheumatology and Immunology, Peking University Third Hospital, Beijing 100191, China; zhao-jinxia@163.com

**Keywords:** isolated antibody positive, hydroxychloroquine, perinatal outcomes, pregnancy, non-specific auto-antibody

## Abstract

**Objective:** This study aimed to investigate the effect of hydroxychloroquine (HCQ) application during pregnancy on perinatal outcomes in cases of combined non-specific auto-antibodies. **Methods**: A retrospective cohort study was carried out. Cases of pregnancy combined with isolated auto-antibody positivity at Peking University Third Hospital from 2016 to 2020 were included. HCQ use during pregnancy was defined as the primary exposure. The impact of HCQ on perinatal outcomes was explored through univariate and multivariate analyses, and stratified analyses of its effects were conducted according to prophylactic anticoagulation use and medication duration. **Results**: A total of 338 cases were included, accounting for 39.62% (338/853) of pregnancies with autoimmune abnormalities during the same period. Univariate analysis of the overall population showed that HCQ use during pregnancy was associated with a significantly lower incidence of pre-eclampsia (9.13% vs. 25.53%), early-onset pre-eclampsia (1.37% vs. 10.08%), and small for gestational age (SGA) (12.07% vs. 22.88%). In the subgroup without anticoagulation, both multivariate and univariate analyses revealed that HCQ was associated with markedly lower rates of pre-eclampsia (0% vs. 36.67%, *p* = 0.004), early-onset pre-eclampsia (0% vs. 15.00%, *p* = 0.046), and SGA (0% vs. 28.33%, *p* = 0.006), and a significantly longer pregnancy gestational age with a higher birth weight. The timing of HCQ initiation showed no significant impact on adverse pregnancy outcomes. **Conclusions**: HCQ use during pregnancy is associated with favorable perinatal outcomes among women with isolated non-specific auto-antibody positivity, especially in those not receiving anticoagulation. Strengthened clinical evaluation and careful risk–benefit assessment are warranted to avoid unnecessary interventions.

## 1. Introduction

HCQ, with its multilayered immunomodulatory effects [1], is an important drug for the control of autoimmune diseases (AIDs), such as systemic lupus erythematosus (SLE), Sjogren’s syndrome (SS), antiphospholipid syndrome (APS), and undifferentiated connective tissue disease (UCTD), during pregnancy [2]. Moreover, it is valuable for improving adverse perinatal outcomes, such as pre-eclampsia, in people with these diseases [3,4,5].

HCQ is not currently used as first-line treatment during pregnancy in cases of combined or isolated non-organ specific auto-antibody positivity. Whether HCQ can also improve perinatal outcomes in this population is controversial. With the recent increase in the number of cases of empirical use of HCQ in the abnormal autoimmune population, we designed this study based on our understanding of the pharmacological mechanisms of HCQ, pregnancy immune status, changes in coagulation, and the pathogenesis of placental dysfunction-related diseases. The study was designed to summarize the effects of HCQ on the prevention and treatment of pre-eclampsia and other disorders and to investigate the effects of HCQ on adverse perinatal outcomes associated with placental dysfunction.

## 2. Methods

### 2.1. Inclusion Criteria

The data was extracted from a database on pregnancy complicated with autoimmune disorders previously established by our team [6]. At that time, there was significant heterogeneity in the clinical management of such patients, and the valuable retrospective data provided us with the opportunity to analyze the effects of different medications.

All cases of pregnant women with isolated non-specific auto-antibody positivity who were admitted and delivered in the obstetrics department of Peking University Third Hospital from 1 January 2016 to 31 December 2020 were included. Details on hospitalization, complete case files, and clear diagnostic information were obtained.

### 2.2. Exclusion Criteria

The exclusion criteria were as follows: Pregnancy loss before 14 weeks of gestation (since the study was based on obstetric inpatient medical records, and data on early pregnancy events were not captured in outpatient/emergency department records). Pregnancies not delivered at our hospital or with incomplete medical records were excluded. Abortion or mid-term induction of labor unrelated to disease, which refers to the abandonment of pregnancy due to fetal malformation, social factors, etc.

### 2.3. Case Screening Process

Within the target time frame, cases in the electronic medical record system were initially screened in the following order: “autoimmune disease”, “connective tissue disease”, “auto-antibody positive”, “lupus anticoagulant positive”, “adverse pregnancy and delivery history” and “recurrent miscarriage or habitual abortion” by diagnostic keywords or ICD coding standard diagnostic terms. The final selection of people with auto-antibody positivity was completed based on the documentation of symptoms, physical examination, and ancillary examination in the case data.

Cases that did not meet the diagnostic criteria for each AID and did not have signs or symptoms of connective tissue disease were classified using the following non-specific auto-antibody panels, including three groups: ① Antinuclear antibody (ANA) profile [ANA, anti-double-stranded DNA (anti-dsDNA), anti-nucleosome, anti-histone, etc.]; ② antiphospholipid antibody (aPL) profile [anticardiolipin antibody (ACL), anti-β2-glycoprotein I (anti-β2-GP I), lupus anticoagulant (LA), etc.]; and ③ other systemic non-organ-specific autoantibodies [antineutrophil cytoplasmic antibody (ANCA) profile, rheumatoid arthritis-related antibody profile, etc]. Notably, organ-specific autoantibodies were explicitly excluded from our cohort, including thyroid autoantibodies, autoimmune liver disease-related antibodies and type 1 diabetes-related antibodies.

### 2.4. Classification, Grouping, and Stratification Criteria

HCQ use during pregnancy was the primary exposure. The entire study population with HCQ application during pregnancy was designated as the exposure group, while the study population without HCQ application during pregnancy was designated as the non-exposure group.

**Figure 1:**
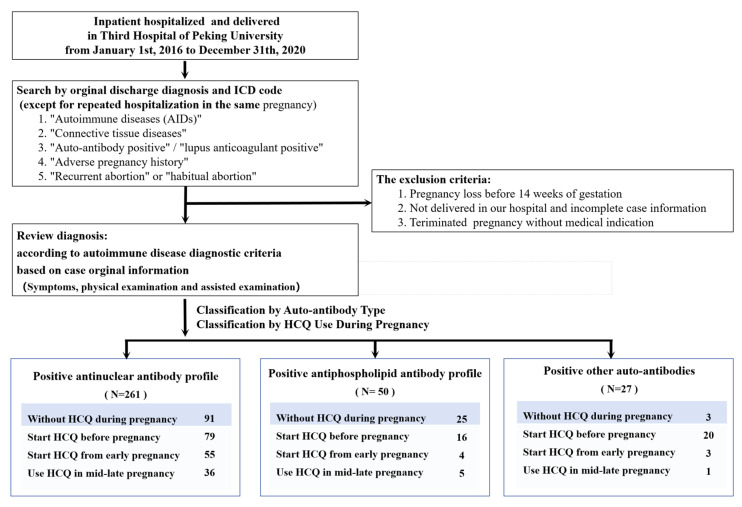
Flow chart for clinical case screening of study population.

The subjects were grouped according to whether they were taking HCQ during pregnancy and the duration of administration. Taking HCQ meant taking HCQ sulfate at a dose of 200–400 mg/day for at least 1 month before pregnancy outcomes occurred. Cases of HCQ use during pregnancy were divided into those starting before pregnancy, those starting in early pregnancy, and those starting in the second and third trimesters according to the duration of administration and the definition of early pregnancy. The case screening and classification processes, as well as the definition of each study population, are shown in Figure 1.

Some of the enrolled patients were on concomitant prophylactic anticoagulation (low-molecular heparin and/or aspirin) to promote improved placental function [7]. To control for the confounding effect of this factor, the effect of HCQ on pregnancy outcomes was explored separately in the “no anticoagulation” and “anticoagulation” populations, which were stratified by whether they were receiving concomitant prophylactic anticoagulation. The impact of HCQ on pregnancy outcomes was explored separately in the “no anticoagulation” and “anticoagulation” populations.

### 2.5. Outcome Indicators

Outcomes were defined according to international guidelines, Chinese clinical consensus and the literature [8,9,10].

Placenta-mediated pregnancy complications (PMPCs) were set as the primary outcomes, focusing on complications linked to underlying placental dysfunction: pre-eclampsia, early-onset pre-eclampsia and small-for-gestational age (SGA). Pre-eclampsia was defined as new onset hypertension (≥140/90 mmHg after 20 weeks’ gestation) plus proteinuria (≥300 mg/24 h or protein/creatinine ≥30 mg/mmol) or end organ involvement (e.g., renal insufficiency, thrombocytopenia). The termination of pregnancy before 34 weeks of gestation due to pre-eclampsia was defined as early-onset pre-eclampsia [10]. For SGA, for fetuses delivered at ≥24 weeks, birth weight below the 10th percentile for gestational age, using a semi-customized fetal growth curve based on the calibration of the Chinese population, was selected by referring to the 2019 Expert Consensus on Fetal Growth Restriction [9].

The secondary outcomes were gestational weeks of delivery, birth weight, neonatal asphyxia, and mid to late pregnancy loss. Neonatal Apgar score of ≤7 at the first minute was defined as neonatal asphyxia to reflect the intrauterine condition of the fetus. The analysis of neonatal asphyxia in this study was performed on live-born babies at all gestational weeks. In this study, birth weight and SGA were analyzed for fetuses delivered at ≥24 weeks of gestational age (newborn, aborted, or stillborn).

### 2.6. Statistical Analysis of Data

Microsoft Excel 2020 was used to collect and organize the data. Statistical analysis of the data was performed on SPSS 26.0 software. The measurement data were described by mean ± standard deviation or median (25th percentile, 75th percentile) in accordance with normality test by using independent sample *t*-test, one-way ANOVA or non-parametric test. Linear regression was used for continuous outcomes; logistic regression was used for binary outcomes. Multivariate regression models were adjusted for variables with significant baseline imbalance between groups. Count data were described by the number of cases (percentage), and chi-square test was used for comparison between groups and logistic regression was used for multifactor analysis. Two-sided *p* < 0.05 were considered statistically significant.

## 3. Results

### 3.1. Patient Characteristics

A total of 338 pregnant women with isolated non-specific auto-antibody positivity were enrolled, accounting for 39.62% (338/853) of pregnancies with autoimmune abnormalities during the same period. The study population included 261 cases with a positive ANA profile, 50 cases with a positive aPL profile, and 27 cases with positive other non-specific auto-antibodies (Figure 1, Table 1).

The rates of pre-eclampsia (14.20%), early-onset pre-eclampsia (4.44%) and SGA (15.71%) were higher than those in normal population. A higher proportion of those who applied HCQ also received prophylactic anticoagulation (91.78% vs. 49.58%) and/or immunomodulatory therapy (49.77% vs. 14.29%) compared with those who did not apply HCQ (*p* < 0.001).

### 3.2. Effect of HCQ Application During Pregnancy on Pregnancy Outcomes

No maternal and fetal abnormalities clearly associated with HCQ occurred at our case follow-up. Univariate analysis suggested that the use of HCQ use was associated with a significantly lower incidence of mid- to late-term pregnancy losses (3.36% vs. 0%, *p* = 0.006), pre-eclampsia (23.53% vs. 9.13%, *p* < 0.001), early-onset pre-eclampsia (10.08% vs. 1.37%, *p* < 0.001), and significantly prolonged the gestational weeks of labor (36.60 ± 5.16 weeks vs. 38.26 ± 2.57 weeks, *p* = 0.001), Table 1.

For the multifactorial analysis of this population, which was stratified by the use of anticoagulation or not, the univariate and multifactorial regression analyses of HCQ and pregnancy outcomes are shown in Table 2 and Table 3. Multivariate regression models were adjusted for variables with a significant baseline imbalance between groups. In the non-anticoagulated population, HCQ during pregnancy significantly reduced the incidence of pre-eclampsia (36.67% vs. 0%, *p* = 0.002) and SGA (28.33% vs. 0%, *p* = 0.042) and significantly prolonged gestational weeks of delivery (36.21 ± 5.23 weeks vs. 39.24 ± 1.23 weeks, *p* = 0.018). It increased birth weight (2756.50 ± 856.08 g vs. 3366.11 ± 394.43 g, *p* = 0.005). No significant association was observed in the anticoagulation group. No consistent favorable trend was observed.

### 3.3. Effect of Different Medication Time Frames on Pregnancy Outcomes in the Study Population

The study population consisted of 219 cases of HCQ application during pregnancy, which were further divided into three groups according to the time frame of HCQ application. The demographic information and clinical characteristics of the study population are shown in Table 4. There was no statistical difference in the incidence of adverse pregnancy outcomes among patients who started medication before pregnancy, middle-to-late pregnancy, or early pregnancy.

## 4. Discussion

Placental dysfunction-related pregnancy complications, such as pre-eclampsia, fetal growth restriction, recurrent early spontaneous abortion, oligohydramnios and placental abruption [8], pose significant threats to maternal and fetal health [4,8]. These complications are characterized by the same pathological mechanisms in the first trimester, including maternal–fetal interface immune disorders, inadequate extravillous trophoblast invasion, and defective uterine spiral artery remodeling [11]. This may also be the basis of pathological pregnancy in women with autoimmune abnormalities [12]. Our recent study on APS has revealed that pathogenic antibodies induce extravillous trophoblast dysfunction, which may contribute to adverse pregnancy outcomes [13].

Clinically, HCQ has been associated with a lower incidence of adverse pregnancy outcomes, such as pre-eclampsia, in AIDs like SLE [14,15], APS [16], and UCTD [2]. HCQ is generally considered safe in pregnancy and lactation for the treatment of rheumatic conditions. The risk of drug-related ocular involvement in patients under routine monitoring was very low [17]. Originally an antimalarial drug, HCQ has evolved into a widely used nonspecific anti-inflammatory agent [2,18,19]. It functions by inhibiting autophagy, regulating toll-like receptors, inflammatory cytokines and complement responses, both systemically and locally in the endometrium [2,18]. Additionally, HCQ exhibits vascular endothelial protection and potential anticoagulant properties [1,19].

For people with positive antibodies, HCQ is not recommended as a first-line drug in current clinical guidelines or consensus on disease, and the benefit of HCQ on pregnancy outcomes is still controversial [20,21]. In this study, we investigated the significance of the use of HCQ during pregnancy on the improvement of pregnancy outcomes in this group.

In the study population, univariate, multivariate, and stratified multifactorial analyses all showed that HCQ application during pregnancy was associated with a lower risk of pre-eclampsia, as shown in Table 1, Table 2 and Table 3. Combined with the univariate pregnancy outcomes analysis, the results of the stratified multifactorial analysis suggested that although the effect of anticoagulant drugs masked the effect of HCQ to some extent [7,22], the comparison with pregnancy outcomes in the non-anticoagulant population still supported a potential link between HCQ use and more favorable placenta-related pregnancy outcomes. This thereby supports a potential mechanism for HCQ in the prevention and treatment of placental dysfunction in the autoimmune abnormal population.

The current drugs for the prevention and treatment of complications associated with placental dysfunction have only focused on antispasmodic, antihypertensive, and anti-thrombosis therapy. Moreover, the limitation of aspirin in the prevention of pre-eclampsia is also common [23,24,25]. Our study suggests a potential favorable association of HCQ use for pregnant patients with non-specific antibodies. It may be a considerable option due to its safety in pregnancy, convenient oral administration operability, and cost-effectiveness.

According to the time frame of HCQ application, as shown in Table 4, the incidence of adverse pregnancy outcomes was not statistically different whether it was started in the first trimester, second and third trimesters of pregnancy, or pre-pregnancy. The main reasons for this finding may be the insufficient cumulative duration of action of HCQ when applied during pregnancy and insufficient time to achieve a stable effect in those who started it in the early and mid-pregnancy stages.

In the pathogenesis of pregnancy complications related to placental dysfunction, there is an excessive state of inflammatory response at the maternal–fetal interface in early pregnancy [26,27,28], especially in the presence of autoimmune abnormalities; effective immunomodulation is of greater significance in early pregnancy, especially before placental formation [2]. However, HCQ is a slow-acting drug, and its effects are cumulative. The onset of effects occurs only after a minimum of 30 days. To ensure a stable drug effect in early pregnancy, it is currently recommended that patients with an indication for HCQ take 200–400 mg/day 3–6 months prior to planned pregnancy [19]. The study population, without a history of AID, mostly due to infertility or adverse obstetric history, start treatment with HCQ only when auto-antibody positivity is detected before planning a pregnancy or in early pregnancy, resulting in a relatively late start of treatment [29].

Our study also identified a lack of standardized clinical management for this group in clinical practice. Overdiagnosis and overmedication of AIDs are common. As shown in Table 1, the group treated with HCQ has more instances of pregnancies, primiparas, and ≥2 previous early pregnancy losses, which suggests that a history of adverse pregnancy and an urgent desire to become pregnant may be one of the reasons why such patients actively accept empirical or even combined medication. In clinical practice, a history of adverse pregnancy and delivery, or even infertility and repeated transplant failure in assisted reproduction, increases the financial and psycho-spiritual pressure on patients and their families, leading to an urgent desire to become pregnant. The clinical diagnosis of AIDs is also affected by the lack of symptom observation, abnormal immunological indicators, and standardized follow-up and treatment.

In addition, the study population was described as “controversial for HCQ use” during pregnancy. The combination of drugs should be used with greater caution. A comparison of the two groups showed that a significantly higher proportion of cases with HCQ were receiving concomitant prophylactic anticoagulation and/or immunomodulation therapy. Glucocorticoids and other immunosuppressive treatments in this population should be of concern, and the population needs to be alert to the potential risks of the associated co-medications.

In view of the unstandardized diagnosis of AIDs in pregnancy and the existence of over-indications for the use of drugs during pregnancy, the relevant data (such as specialist outpatient records, obstetric outpatient records, inpatient records and records of multidisciplinary discussions or specialist consultations) in the electronic medical record system of the enrolled cases were retraced one by one in this study. In addition, the diagnosis and classification of the disease were rechecked in accordance with the recorded symptoms and physical and auxiliary examination results of the patients. This process enhanced the accuracy and reliability of study classification. However, due to the limitations of the retrospective case data and the urgent desire of some patients to prepare for pregnancy, the timeframe for observation of relevant symptoms and abnormal laboratory indicators was insufficient and the review of the diagnostic process may result in a very small proportion of autoimmunity that is still in a sub-clinical stage not being identified and diagnosed.

This study has several limitations. First, early pregnancy outcomes (e.g., biochemical pregnancy, early spontaneous abortion) were not included, as data were derived from the obstetric inpatient medical record system (outpatient-managed early pregnancy events were not captured). This exclusion may introduce selection bias and underestimate the overall burden of adverse pregnancy outcomes in this population. Second, we pooled patients with different non-specific auto-antibody subtypes (ANA, aPL, ANCA profiles) into a single group. Despite all patients not meeting AID/CTD diagnostic criteria, these subtypes have distinct pathogenic mechanisms and may exert differential effects on placental function and pregnancy outcomes. This heterogeneity may mask subtype-specific associations and limit the interpretability of our findings; future studies should explore HCQ’s role in individual auto-antibody subtypes. Third, our case identification strategy (relying on obstetric inpatient records and screening via adverse pregnancy history-related keywords) introduced inherent selection bias, as the cohort was enriched with high-risk patients. Consequently, our findings may not be generalizable to women with isolated auto-antibody positivity but no prior adverse pregnancy outcomes. Fourth, as a retrospective observational study, confounding-by-indication and unmeasured confounding could not be fully excluded. Although we performed stratification and multivariable adjustment for known confounders, residual or unknown confounding factors may still have influenced the observed associations, which warrants cautious interpretation of the results.

In conclusion, this study uniquely focuses on auto-antibody positive populations rather than just confirmed AID cases, highlighting a potential favorable association of HCQ use with placenta-mediated pregnancy complications, supporting further study of HCQ for reducing pre-eclampsia risk in this population. Moving forward, prospective multicenter cohort studies with larger sample sizes and standardized HCQ initiation protocols are urgently needed. These future studies should aim to validate our current findings and further explore the long term efficacy and safety of HCQ in autoimmune abnormal populations, thereby providing more robust evidence for clinical decision making.

## Figures and Tables

**Table 1:** Demographic characteristics and clinical features of the study population.

	Without HCQ 119 (35.21%)	Take HCQ 219 (64.79%)	Z/X^2^	*p*
**Demographic and medical history information**				
Age (years)	34.40 ± 4.73	33.79 ± 3.69	1.236	0.218
Gravity (times)	2.00 [1.00, 3.00]	3.00 [2.00, 3.00]	2.235	0.025
Primiparas [n (%)]	87 (73.11)	195 (89.04)	14.158	<0.001
History of early pregnancy loss ≥ 2 times [n (%)]	23 (19.33)	81 (36.99)	11.287	0.001
Obesity [n (%)]	15 (12.61)	7 (3.20)	11.216	0.001
History of pre-eclampsia [n (%)]	9 (7.56)	9 (4.11)	1.824	0.177
**Current pregnancy**				
Embryo transfer [n (%)]	25 (21.01)	51 (23.29)	0.230	0.632
Multiple pregnancy [n (%)]	6 (5.04%)	13 (5.94)	0.116	0.733
Combined kidney disease [n (%)]	12 (10.08)	10 (4.57)	3.858	0.050
Chronic hypertension [n (%)]	33 (27.73)	61 (27.85)	0.001	0.981
Pre-gestational diabetes mellitus [n (%)]	8 (6.72)	1 (0.46)	11.680	0.001
Gestational diabetes mellitus [n (%)]	25 (21.01)	51 (23.29)	0.230	0.632
Prophylactic anticoagulation [n (%)]	59 (49.58)	201 (91.78)	77.354	<0.001
Immunomodulatory/immunosuppressant [n(%)]	17 (14.29)	109 (49.77)	41.525	<0.001
**Perinatal outcomes**				
Mid- to late-term pregnancy loss [n (%)]	4 (3.36)	0 (0)	7.450	0.006
Pre-eclampsia [n (%)]	28 (23.53)	20 (9.13)	13.116	<0.001
Early-onset pre-eclampsia [n (%)]	12 (10.08)	3 (1.37)	13.806	<0.001
Gestational week of delivery (weeks)	36.60 ± 5.16	38.26 ± 2.57	3.301	0.001
Birth weight (g)	2894.24 ± 764.07	3022.54 ± 637.37	1.568	0.119
Small for gestational age [cases/total, %]	27/118 (22.88)	28/232 (12.07)	6.904	0.009
Neonatal asphyxia [cases/total,%]	2/115 (1.74)	4/228 (1.75)	#	>0.99

#: Fisher’s exact test.

**Table 2:** Analysis of perinatal outcomes in the study population without anticoagulants (N = 78).

	Without HCQ	Application of HCQ	*p*	RD/MD (95% CI)	a*p*	aRD/MD (95% CI) *
	n = 60	n = 18				
Mid- to late-term pregnancy loss [n (%)]	3 (5.00)	0 (0)	0.333	−5.000 (−15.200, 5.200)	0.449	−3.964 (−14.160, 6.232)
Pre-eclampsia [n (%)]	22 (36.67)	0 (0)	0.002	−36.667 (−59.220, −14.113)	0.004	−34.745 (−57.720, −11.770)
Early-onset pre-eclampsia [n (%)]	9 (15.00)	0 (0)	0.081	−15.000 (−31.711, 1.711)	0.046	−17.446 (−34.290, −0.601)
Gestational week of delivery (weeks)	36.21 ± 5.23	39.24 ± 1.23	<0.001	3.024 (0.576, 5.471)	0.020	3.126 (0.554, 5.699)
Birth weight (g)	2756.50 ± 856.08	3366.11 ± 394.43	<0.001	609.611 (200.334, 1018.888)	0.001	698.010 (284.850, 1111.170)
Small for gestational age [cases/total, %]	17/60 (28.33)	0/18 (0)	0.026	−28.333 (−49.423, −7.244)	0.006	−31.156 (−52.875, −9.437)
Neonatal asphyxia [cases/total, %]	0/57 (0)	0/18 (0)	1.000	0 (0,inf)	1.000	0 (0,inf)

RD, Risk Difference for binary variable; MD, Mean Difference for continuous variable; *, corrected for statistically significant baseline variables in the univariate analysis.

**Table 3:** Analysis of perinatal outcomes in the study population with anticoagulants (N = 260).

	Without HCQ	Application of HCQ	*p*	RD/MD (95% CI)	a*p*	aOR/MD (95% CI) *
	n = 59	n = 201				
Mid to late term pregnancy loss [n (%)]	1 (1.69)	0 (0)	0.065	−1.695 (−3.486, 0.097)	0.208	−1.197 (−3.056, 0.663)
Pre-eclampsia [n (%)]	6 (10.17)	20 (9.95)	0.961	0.976 (0.393, 2.780)	0.445	1.514 (0.554, 4.803)
Early-onset pre-eclampsia [n (%)]	3 (5.08)	3 (1.49)	0.128	0.283 (0.051, 1.564)	0.776	0.749 (0.102, 6.727)
Gestational week of delivery (weeks)	36.99 ± 5.10	38.17 ± 2.64	0.018	1.186 (0.212, 2.160)	0.592	0.273 (−0.724, 1.269)
Birth weight (g)	3036.72 ± 631.75	2993.64 ± 646.02	0.651	−43.079 (−229.655, 143.496)	0.464	−73.737 (−270.901, 123.427)
Small for gestational age [cases/total, %]	10/58 (17.24)	28/214 (13.08)	0.419	0.723 (0.337, 1.657)	0.372	0.676 (0.293, 1.667)
Neonatal asphyxia [cases/total, %]	2/58 (3.45)	4/210 (1.90)	0.488	0.544 (0.103, 3.994)	0.426	0.469 (0.074, 3.788)

RD, Risk Difference for binary variable; MD, Mean Difference for continuous variable; *, corrected for statistically significant baseline variables in the univariate analysis.

**Table 4:** Analysis of hydroxychloroquine and perinatal outcomes in study population with HCQ.

	Start HCQ Before Pregnancy 115 (52.51%)	Start HCQ from Early Pregnancy 62 (28.31%)	Start HCQ from Mid-Late Pregnancy 42 (19.18%)	F/X^2^	*p*
**Demographic and medical history information**					
Age (years)	33.98 ± 3.76	33.18 ± 3.50	34.14 ± 3.76	1.205	0.302
Gravity (times)	3 [2,3]	3 [2,4]	2 [1,3]	3.672	0.160
Primiparas [n (%)]	108 (93.91)	53 (85.48)	34 (80.95)	6.417	0.040
History of early pregnancy loss ≥ 2 times [n (%)]	50 (43.48)	22 (35.48)	9 (21.43)	6.501	0.039
Obesity [n (%)]	3 (2.61)	2 (3.23)	2 (4.76)	#	0.874
History of pre-eclampsia [n (%)]	3 (2.61)	3 (4.84)	3 (7.14)	#	0.354
**Current pregnancy**					
Embryo transfer [n (%)]	31 (26.96)	10 (16.13)	10 (23.81)	2.651	0.266
Multiple pregnancy [n (%)]	8 (6.96)	4 (6.45)	1 (2.38)	1.195	0.550
Combined kidney disease [n (%)]	2 (1.74)	3 (4.84)	5 (11.90)	7.310	0.026
Chronic hypertension [n (%)]	1 (0.87)	0 (0)	0 (0%)	#	0.428
Pregestational diabetes mellitus [n (%)]	29 (25.22)	17 (27.42)	15 (35.71)	0.908	0.635
Gestational diabetes mellitus [n (%)]	31 (26.96)	10 (16.13)	10 (23.81)	2.651	0.266
**Other medications during pregnancy**					
Prophylactic anticoagulation [n (%)]	111 (96.52)	58 (93.55)	32 (76.19)	17.216	<0.001
Immunomodulatory / immunosuppressant [n (%)]	76 (66.09)	26 (41.94)	7 (16.67)	32.180	<0.001
**Perinatal outcomes**					
Mid to late term pregnancy loss [n (%)]	0 (0)	0 (0)	0 (0%)	#	1.000
Pre-eclampsia [n (%)]	9 (7.83)	7 (11.29)	4 (9.52%)	0.592	0.744
Early-onset pre-eclampsia [n (%)]	0 (0)	2 (3.23)	1 (2.38%)	#	0.105
Gestational week of delivery (weeks)	38.47 ± 2.06	38.34 ± 2.64	37.56 ± 3.50	2.004	0.137
Birth weight (g)	3034.88 ± 545.41	3035.30 ± 622.65	2967.67 ± 875.49	0.194	0.824
Small for gestational age [cases/total, %]	14/123 (11.38)	8/66 (12.12)	6/43 (13.95)	0.199	0.905
Neonatal asphyxia [cases/total, %]	2/122 (1.64)	1/65 (1.54)	1/41 (2.44)	#	>0.99

#: Fisher’s exact probability method.

## Data Availability

The data that support the findings of this study are available from Third Hospital of Peking University but restrictions apply to the availability of these data, which were used under license for the current study, and so are not publicly available. However, data are available from the corresponding author on reasonable request and with permission of Third Hospital of Peking University.
